# Spatiotemporal cortical dynamics for visual scene processing as revealed by EEG decoding

**DOI:** 10.3389/fnins.2023.1167719

**Published:** 2023-11-01

**Authors:** Taiki Orima, Isamu Motoyoshi

**Affiliations:** ^1^Department of Life Sciences, The University of Tokyo, Tokyo, Japan; ^2^Japan Society for the Promotion of Science, Tokyo, Japan

**Keywords:** natural scene perception, EEG, brain decoding, EEGNet, Grad-CAM

## Abstract

The human visual system rapidly recognizes the categories and global properties of complex natural scenes. The present study investigated the spatiotemporal dynamics of neural signals involved in visual scene processing using electroencephalography (EEG) decoding. We recorded visual evoked potentials from 11 human observers for 232 natural scenes, each of which belonged to one of 13 natural scene categories (e.g., a bedroom or open country) and had three global properties (naturalness, openness, and roughness). We trained a deep convolutional classification model of the natural scene categories and global properties using EEGNet. Having confirmed that the model successfully classified natural scene categories and the three global properties, we applied Grad-CAM to the EEGNet model to visualize the EEG channels and time points that contributed to the classification. The analysis showed that EEG signals in the occipital electrodes at short latencies (approximately 80 ~ ms) contributed to the classifications, whereas those in the frontal electrodes at relatively long latencies (200 ~ ms) contributed to the classification of naturalness and the individual scene category. These results suggest that different global properties are encoded in different cortical areas and with different timings, and that the combination of the EEGNet model and Grad-CAM can be a tool to investigate both temporal and spatial distribution of natural scene processing in the human brain.

## 1. Introduction

It is widely known that the human visual system rapidly discriminates complex natural scenes (Thorpe et al., 1996; Fabre-Thorpe et al., 2001; Oliva and Torralba, 2001; VanRullen and Thorpe, 2001), perceives the content of visual scene even with a short time presentation (Potter, 1975; Intraub, 1981; Greene and Oliva, 2009; Peelen et al., 2009) and utilizes perceived information to judge the surrounding environment and for spatial navigation. This rapid perception is thought to be based on information obtained by a glance at a natural scene, which precedes the perception of individual objects or detailed features within the scene. Such information is often referred to as gist, which has been successfully formulated as a relatively global image feature such as the spatial envelope (Oliva and Torralba, 2001; Torralba and Oliva, 2003; Groen et al., 2012). Specifically, the spatial envelope is a low-order feature designed to provide a good estimate of the degrees of important indicators to characterize a class of natural scenes, such as naturalness, openness, roughness, expansion, and ruggedness (Oliva and Torralba, 2001). According to the previous studies that investigated behavioral responses to natural scene images, some of these indicators and natural scene categories can be discriminated with high accuracy and short reaction time even when the visual stimuli are briefly presented (Joubert et al., 2007) followed by mask patterns (Bacon-Macé et al., 2005; Greene and Oliva, 2009; Peelen et al., 2009). However, these behavioral data have various factors beyond visual processing of the target scene itself, such as the properties of the backward masking effect (Breitmeyer et al., 2006) and the decision process for response selection (Shadlen and Kiani, 2013; Ratcliff et al., 2016). Analyzing the brain activities for natural scene images may enable us to understand the dynamics of scene processing in humans more directly.

Neural mechanisms of scene perception in the human brain have been most extensively investigated through functional magnetic resonance imaging (fMRI) (Groen et al., 2017). Comparisons of blood oxygenation level dependent signals between visual stimuli having specific characteristics, such as scenes and faces, have revealed scene-selective regions that are important for the perception of natural scenes. The parahippocampal place area is located from the posterior part of the parahippocampal gyrus to the anterior part of the spindle gyrus and has been identified as a region that shows preference for buildings (e.g., Aguirre et al., 1998; Epstein and Kanwisher, 1998). The retrosplenial complex, which is active against mental images of the scene, and the occipital place area, which shows preference for the boundaries of the environment in navigation, have also been identified as scene-selective regions (Nakamura et al., 2000; O'Craven and Kanwisher, 2000; Groen et al., 2016; Julian et al., 2016; Bonner and Epstein, 2018; Epstein and Baker, 2019). These findings suggest that multiple areas in the human brain process different types of information from natural scene images. However, because of the low temporal resolution of fMRI, the cited work could not specify the early neural activities corresponding to rapid natural scene processing, which is probably based on image features as suggested by a number of psychophysical and computational studies (Schyns and Oliva, 1994; Baddeley, 1997; Oliva et al., 1999; Oliva and Torralba, 2001; Gaspar and Rousselet, 2009).

Meanwhile, the temporal dynamics of neural processing underlying natural scene recognition have been investigated through electroencephalography (EEG). A recent study showed that differences in the global information of natural scenes evoked different visual evoked potentials (VEPs) (Harel et al., 2016; Hansen et al., 2018). Another line of research has focused on the hierarchical neural processing of image features that are important for scene recognition. Focusing on a lower-order feature called contrast energy and a higher-order feature called the spatial coherence of natural scene images, Groen et al. (2013) showed that the modulation of EEG by contrast energy terminated in 100–150 ms, whereas the modulation by spatial coherence lasted up to 250 ms. Greene and Hansen (2020) investigated the relationship between event related potentials (ERPs) and a wide range of features from lower to higher order (i.e., features ranging from simple texture statistics of natural scenes to convolutional neural network (CNN) features) and found differences in the encoding process for each feature. Referring to a large body of evidence suggesting that the important features for the instantaneous perception of natural scenes are relatively global (Oliva and Torralba, 2001; Greene and Oliva, 2009; Groen et al., 2013; Kauffmann et al., 2014; Ramkumar et al., 2016), it has been suggested that the natural scene encoding process at an early stage can be investigated using EEG (Ghebreab et al., 2009; Scholte et al., 2009; Võ and Wolfe, 2013; Groen et al., 2016). However, these studies did not mainly step into the spatial distribution of the scene-related neural activity over the cortex maybe because of the low spatial resolution of EEG.

Although various psychophysical and neurophysiological approaches have been adopted to examine the perception of natural scenes, it remains unclear, both spatially and temporally, what part of the brain activity at short latencies contributes to the classification of natural scene categories and global properties. As described in the previous studies, this perception is partially supported by the global information, which may be reflected in the VEPs. If it is the case, the VEPs for natural scene images can classify natural scene categories and global properties of the corresponding images, and we can investigate what part of the brain and what times of the EEG signal contribute to the classification. To test this hypothesis, in the present study, we conducted experiments to investigate the spatiotemporal development of neural information related to scene categories (e.g., a bedroom and forest) and fundamental global properties (i.e., the degrees of naturalness, openness, and roughness) using VEPs. We trained the EEGNet model (Lawhern et al., 2018), which was a CNN model that predicted the natural scene categories and global properties (degrees of naturalness, openness, and roughness) of corresponding natural scene images to inputting VEPs, and visualized the VEP time points and EEG channels that contributed to the classification using Grad-CAM (Selvaraju et al., 2017). These analyses showed that the corresponding natural scene categories and global properties could be classified from simple VEPs at a statistically significant level, and they visually revealed that the different time points and EEG channels contributed to different classification classes. In particular, we found that early-latency (approximately 80 ~ ms) VEPs contributed to the openness classifications, and that both frontal and occipital electrodes contributed to the natural scene category and naturalness classification. These results suggest that different global properties, which have been considered to be important for natural scene recognition, are processed in different cortical areas, and that their localization has already occurred within a short latency of ~100 ms. In addition, these findings further support the idea that the combination of the EEGNet and Grad-CAM can carve out the dynamic neural processing of complex natural images even by using EEG with poor spatial resolution.

## 2. Materials and methods

We measured VEPs for various natural scene images and constructed an EEGNet model using the VEPs as input. We examined how accurately the model classified the natural scene categories and global properties of corresponding images. We then applied Grad-CAM to the EEGNet models to visualize the time points and EEG channels of the VEPs that contributed to the classification.

### 2.1. Observers

Twelve naïve students participated in the experiment. All participants had normal or corrected-to-normal vision. All experiments were conducted in accordance with the guidelines of the Ethics Committee for experiments on humans at the Graduate School of Arts and Sciences, The University of Tokyo. All experiments were conducted in accordance with the Declaration of Helsinki. All participants provided written informed consent. One participant was excluded from the following analyses because their EEG data were deficient, that is, the number of recorded triggers were smaller than expected due to a machinery problem.

### 2.2. Apparatus

Visual stimuli were generated by a personal computer (HP Z2 Mini G4 Workstation) and presented on a 24-inch gamma-corrected liquid-crystal display (BenQ XL2420T) with a refresh rate of 60 Hz and a spatial resolution of 1.34 min/pixel at a viewing distance of 100 cm.

### 2.3. Stimuli

**Figure 1:**
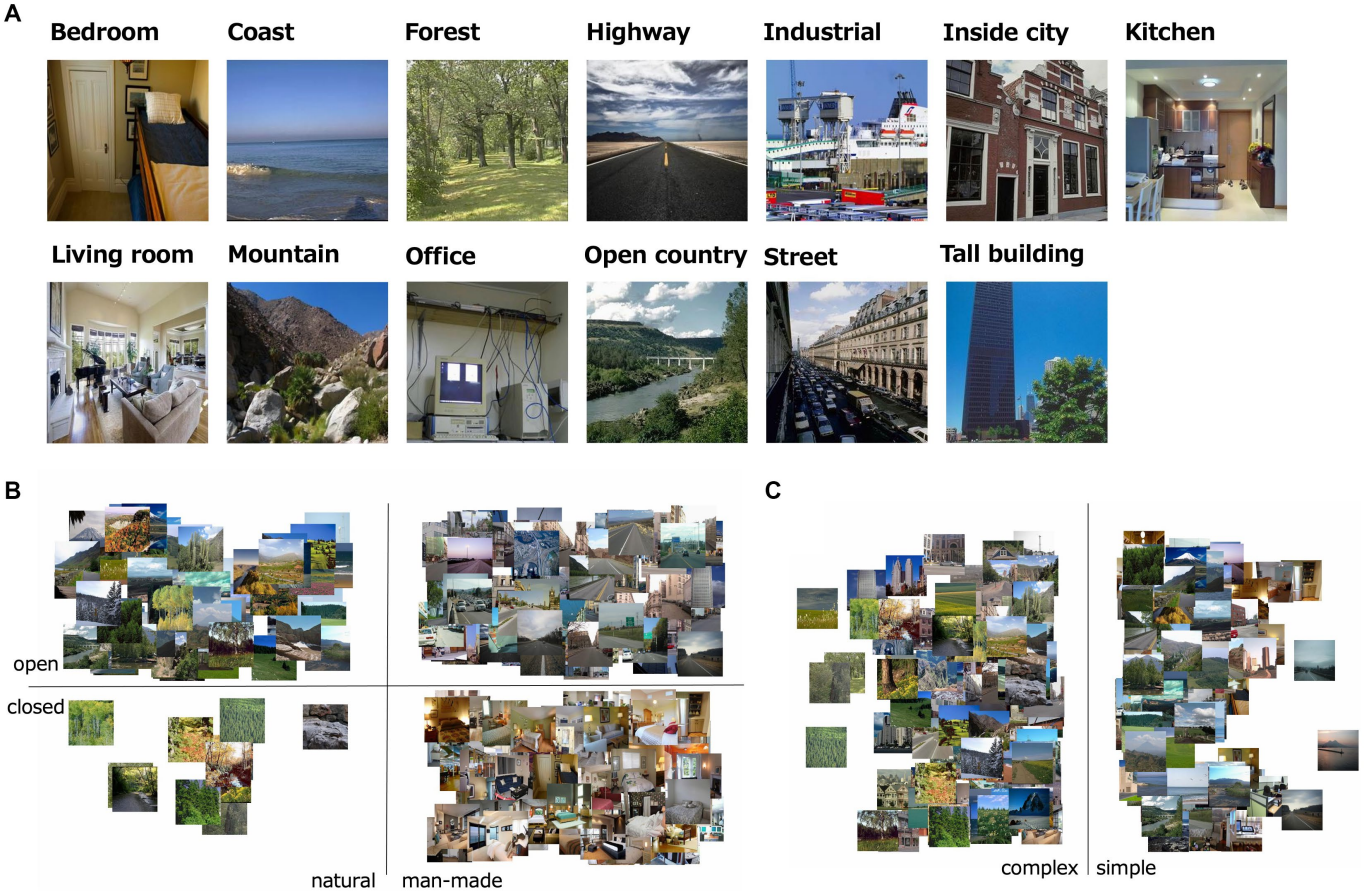
**(A)** Examples of stimuli used in the experiment; i.e., one image from each of the 13 natural scene categories. **(B)** The distribution of naturalness, openness 0/1 values of all the visual stimulus. **(C)** The distribution of roughness values of all the visual stimulus.

The visual stimuli were 232 natural scene images, which were comprised of 5.7 deg × 5.7 deg (256 × 256 pixels; Figure 1). All images were collected via the Internet from the SUN and Places 365 databases (Xiao et al., 2010; Zhou et al., 2014). We assumed these natural images were taken with the gamma of 2.0, and loaded with the gamma of 0.5. All images were classified into one of 13 natural scene categories identified as important in previous studies: offices, kitchens, living rooms, bedrooms, industrial scenes, tall buildings, city scenes, streets, highways, coasts, open country, mountains, and forests (Oliva and Torralba, 2001; Lazebnik et al., 2006; Alameer et al., 2016).

### 2.4. EEG recording procedures

EEG experiments were conducted in a shielded dark room. In each session, 232 natural scene images were presented once in random order. Each image was presented for 500 ms, after which a uniform background blank of 27 cd/m^2^ was presented for approximately 750 ms, which was necessary for brain responses to settle down in the preliminary experiment. Participants observed the stimuli foveally through steady fixation on a small black dot that appeared at the center of the screen. EEG recordings were made while the participants observed the visual stimuli. Participants’ eye movements were controlled by pre-experiment instruction (c.f., Orima and Motoyoshi, 2021). Seventeen sessions were conducted in the experiment, and each image was presented 17 times in total for each participant.

### 2.5. EEG data preprocessing

EEG data were acquired from 19 electrodes (Fp1, Fp2, F3, F4, C3, C4, P3, P4, O1, O2, F7, F8, T7, T8, P7, P8, Fz, Cz, and Pz) in accordance with the international 10–20 system at a sampling rate of 1,000 Hz (BrainVision Recorder, BrainAmp amplifier, EasyCap; Brain Products GmbH). The impedance of each electrode was kept below 5 kΩ. An additional electrode, located between Fz and AFz, was used as the ground. In addition, all electrodes were referenced to another electrode located between Fz and Cz, and all electrode data were re-referenced offline using the average of all electrodes. The recorded EEG data were filtered by a 0.5–40 Hz bandpass filter and divided into epochs of −0.4–0.8 s from the stimulus onset. Baseline correction was performed using the data for −100–0 ms from the stimulus onset as a baseline. The eye movements were removed through independent component analysis and the epochs including abnormal amplitude (exceeding the range from −75 to 75 μV) were rejected to remove epochs with eye blinks.

### 2.6. Training the EEGNet model

EEGNet is a CNN model that treats EEG data as two-dimensional data of time points × EEG channels as input (Lawhern et al., 2018; Lotte et al., 2018). Previous studies have shown that EEGNet performs well in EEG decoding, and because it convolves both in time and in space, it is said to be able to capture the spatiotemporal properties of EEG data (Wakita et al., 2021). Grad-CAM has been used to visualize the portion of inputs that contribute to classification in deep neural network models for object recognition (Selvaraju et al., 2017). In the present study, not only to classify the characteristics of visual stimuli from VEPs but to understand the spatiotemporal portions that contributed to the classification, we trained an EEGNet model to classify corresponding natural scene categories and global properties, and applied Grad-CAM to the EEGNet model to visualize the classification.

**Figure 2:**
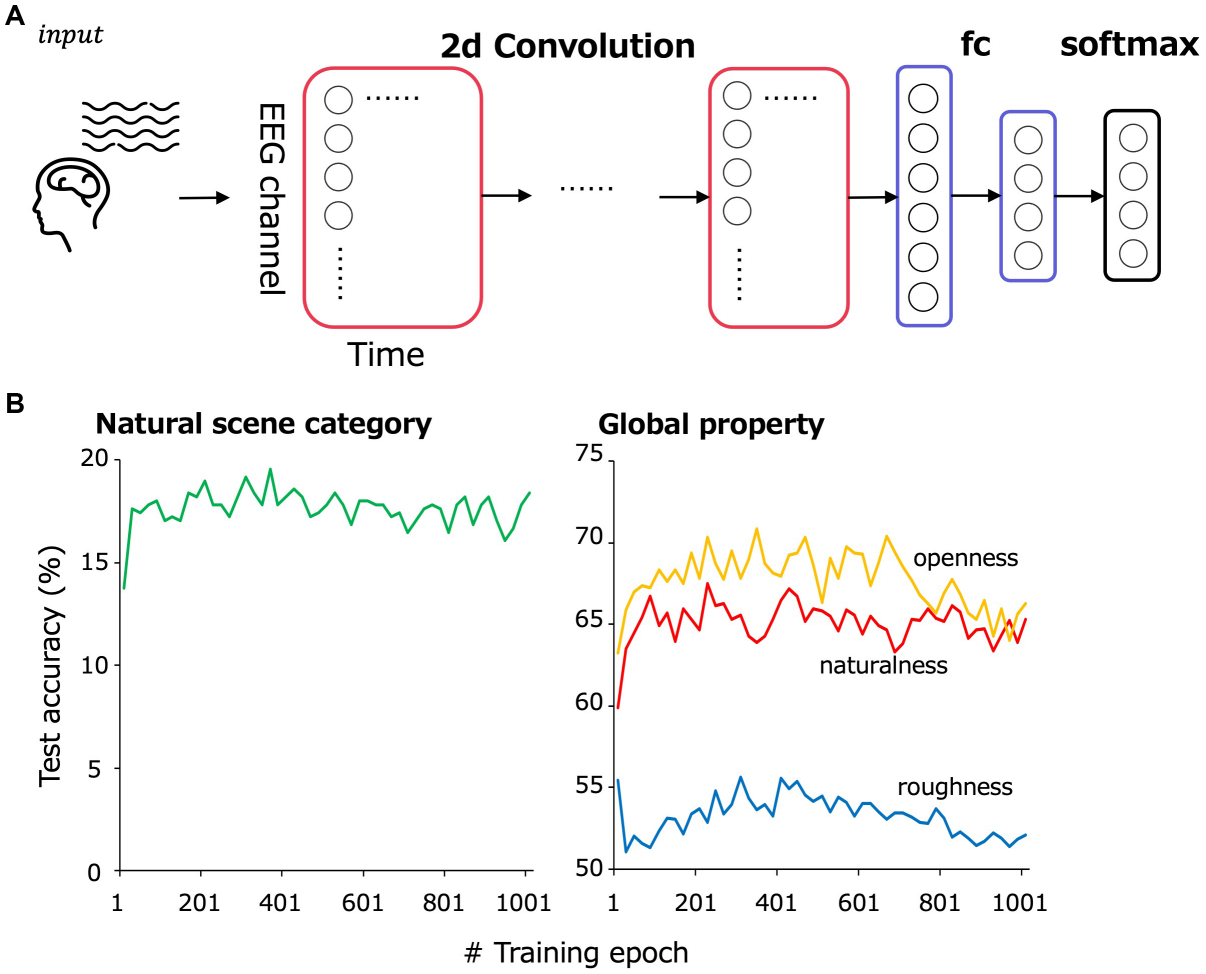
**(A)** Model overview. EEG data were input as a two-dimensional array comprising 17 EEG channels × 500 time points to the convolutional layers. The convolutional layers were followed by a fully connected layer and then a softmax layer to classify natural scene categories and global properties. **(B)** The test accuracy transition of the classification in the cross-validation set 1.

Figure 2A is an overview of the EEGNet model. Following a previous study (Lawhern et al., 2018), EEG data were input as two-dimensional data of time points × EEG channels and trained to classify 13 natural scene categories of the corresponding visual stimuli to the input VEPs. The 232 images were split into training and testing data such that they were almost equally divided within each natural scene category. We performed 5-fold cross validation to secure generalizability of the EEGNet models.

The preprocessed EEG data from 1 to 500 ms of the stimulus onset for 17 electrodes (F3, F4, C3, C4, P3, P4, O1, O2, F7, F8, T7, T8, P7, P8, Fz, Cz, and Pz) in the international 10–20 system were treated as 17 × 500 matrix data. From ~187 samples (11 observers × 17 repetition, some of them were rejected by the preprocess), 30 to 35 samples of EEG data corresponding to a single visual stimulus were picked up in random combinations for input to the model and then averaged. Note that each sample for each image, EEG channel and repetition were z-scored to eliminate the effect of the absolute value of each channel. The number of training epochs was set at 361, at which we confirmed that the classification accuracy in the cross-validation set 1 was highest (Figure 2B). The number of averaged VEP used for training per epoch was 3,000. Table 1 shows the detailed architecture of the EEGNet model. The loss for each iteration was calculated using PyTorch’s torch.nn.CrossEntropyLoss.

**Table 1:** Details of the EEGNet architecture.

Block	Layer	# Filters	size	Activation	Options	
	Input		(17, 500)		Replicate padding: (0, 32)	
ConvBlock1	Conv2d	8	(1, 64)			
	BatchNorm2d					
ConvBlock2	Conv2d	16	(1, 1)			
	BatchNorm2d					
	Activation			ELU		
	AvgPool2d		(1, 4)			
	Dropout				*p* = 0.5	
ConvBlock3	Conv2d	16	(1, 16)		Replicate padding: (0, 8)	
	Conv2d	16	(1, 1)			
	BatchNorm2d					
	Activation			ELU		
	AvgPool2d		(1, 8)			
	Dropout				*p* = 0.5	
	Fully connected				nn.Linear: 4,080 → *N*	
	Softmax				*N*: number of classes	

Besides training the EEGNet model to classify the VEPs into corresponding 13 natural scene categories, we also trained the EEGNet model to classify the VEPs according to global properties that characterize natural scenes, namely the degrees of naturalness, openness, and roughness (Oliva and Torralba, 2001). Naturalness (natural/man-made) had a predefined 0/1 value indicating whether each natural scene was mainly composed of natural or man-made objects. Openness (open/closed) also had a predetermined 0/1 value, indicating whether each natural scene was open or closed. Roughness (simple/complex) was considered to correspond to the ‘complexity’ of the scene (Oliva and Torralba, 2001). In the present study, the slope of the power spectrum of each image, which is related to roughness, was calculated and binarized around its median value to give the roughness of each image (Oliva and Torralba, 2001). The degrees of expansion and ruggedness were excluded from the present study because they are mainly applied only to man-made and natural scenes, respectively.

The architecture of the EEGNet models is the same as that shown in Table 1, except for the size of the final fully-connected and softmax layer. The number of training epochs was set at 221, 341, 301 for the naturalness, openness, roughness classification, respectively, based on the classification accuracy in the cross-validation set 1 (Figure 2B), and the number of samples used for training in one epoch was set at 3000.

### 2.7. Application of Grad-CAM to the EEGNet models

After the training of the EEGNet models, Grad-CAM was adopted to visualize the contribution to the classification. The average VEPs of each participant’s testing data were input to the trained EEGNet model, and the predicted natural scene category or global property were obtained from each VEP. We then applied Grad-CAM to the trained EEGNet model following a previous study (Selvaraju et al., 2017). The output of the convolutional layer in ConvBlock2 was used as the feature map. Next, the gradient of the score for predicted natural scene category or global property with respect to the feature map activations was computed, and the global average pooling of the feature maps was calculated. A localization map was obtained as the multiplicative product of the feature maps and global average pooling. To adopt only the points that contributed positively to the classification, the localization map was finally passed through a ReLU layer. The localization maps that were obtained for each participant were normalized to relative values according to the minimum and maximum values, and averaged across participants and projected onto a topographical map to visualize the time points and EEG channels that contributed to the classification.

### 2.8. Support vector machine settings

Support vector machines (SVMs) were used for the additional analyses in the discussion. We used the Matlab function ‘fitcecoc’ for the natural scene category classification and ‘fitcsvm’ for the others with default settings to train SVMs, and 5-fold cross validation was performed in the same way as the training of the EEGNet models.

## 3. Results

### 3.1. VEPs

**Figure 3:**
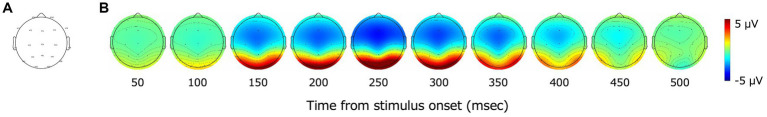
**(A)** The distribution of EEG channels in the present study. Nineteen electrodes (Fp1, Fp2, F3, F4, C3, C4, P3, P4, O1, O2, F7, F8, T7, T8, P7, P8, Fz, Cz, and Pz) in accordance with the international 10–20 system were adopted. **(B)** Topography of grand-average VEPs. Red indicates positive values and blue indicates negative values. A large rise in VEPs was observed after 100 ms from the stimulus onset, mainly in the occipital cortex.

Figure 3A shows the electrode position that we used, and Figure 3B shows the grand-average VEPs for all images from 50 to 500 ms after the stimulus onset. Red indicates positive amplitudes and blue indicates negative amplitudes. VEPs were particularly large for the occipital electrodes (O1, O2). VEPs of the occipital electrodes (O1, O2) began to rise at approximately 100 ms after the stimulus onset. The amplitudes of the VEPs of the occipital electrodes increased again, peaked at approximately 250 ms, and then decreased.

### 3.2. Classification of natural scene categories and global properties using EEGNet models

**Figure 4:**
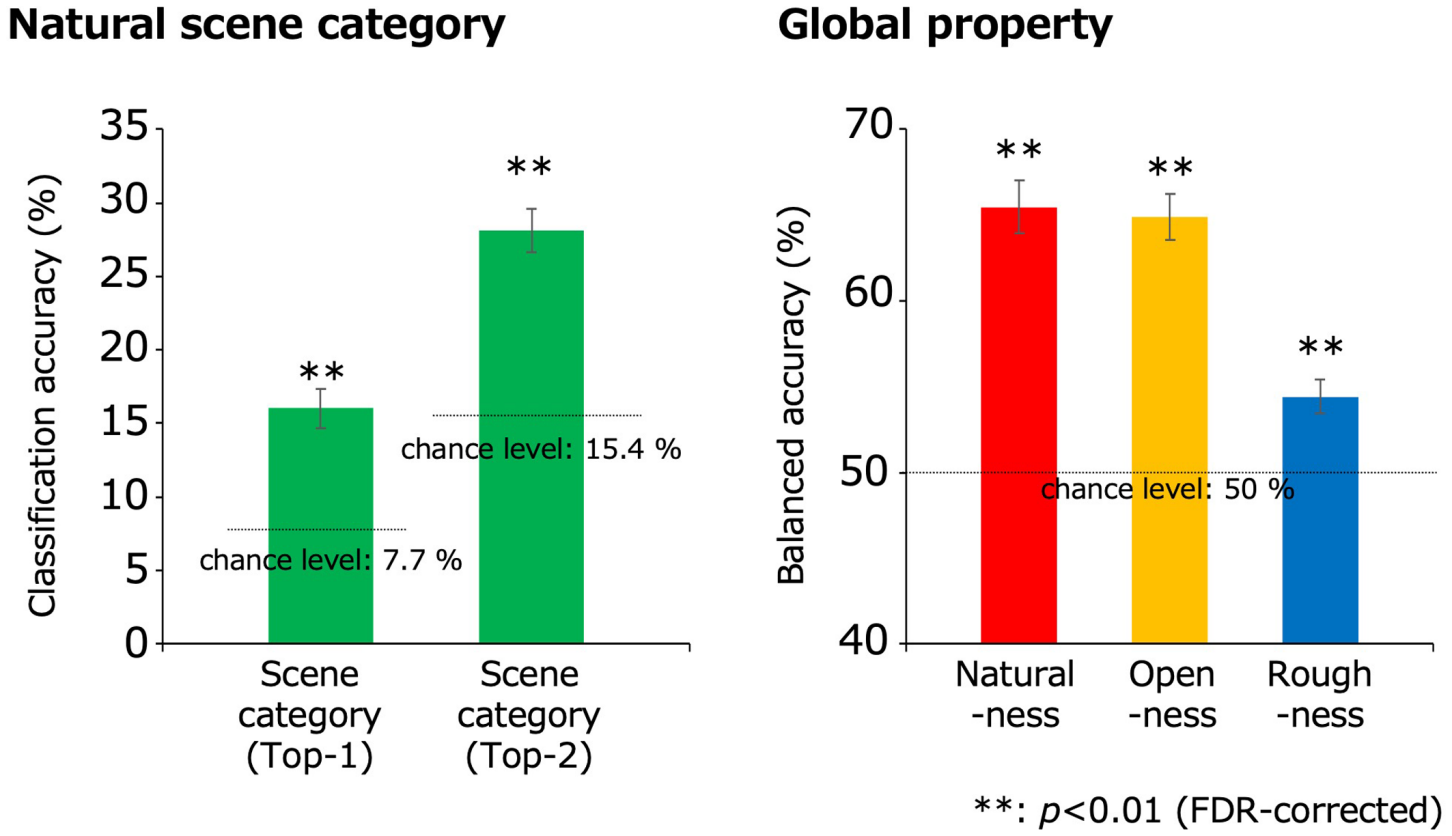
Classification results obtained using the EEGNet models. The green, red, yellow, and blue bars indicate the classification accuracies for the natural scene categories, naturalness, openness, and roughness, respectively, all of which were statistically significant (**p < 0.01, FDR-corrected). In the classification of the natural scene categories, the Top-n classification accuracy is the rate that the correct category was included in the top-n prediction. The dotted lines denote the chance levels. The error bars indicate ± 1 s.e.m. across observers.

Figure 4 shows the classification accuracy of the natural scene categories using the EEGNet model. In each cross-validation set, the VEPs that were assigned to the testing data were averaged within participants and input to the trained EEGNet model, and we obtained the classification accuracy for each participant and cross-validation set. The obtained values of classification accuracy were averaged within participants to obtain a representative classification accuracy for each participant. Finally, these representative values were averaged across participants. The statistical analysis was performed using sample size of 11, which was equal to the number of participants. To address the multiple comparisons, we adopted the Benjamini-Hochberg (BH) false discovery rate (FDR)-correction method (Benjamini and Hochberg, 1995).

The classification accuracy for the 13 natural scene categories was 16.0% (chance level: 1/13 (7.7%); *t*(10) = 5.92; *p* = 1.5 × 10^−4^, two-tailed one-sample *t*-test) and that within the top two categories was 28.1% (chance level: 2/13 (15.4%); *t*(10) = 8.28; *p* = 8.7 × 10^−6^, two-tailed one-sample *t*-test), with both results being statistically significant (*p* < 0.01, FDR-corrected).

Meanwhile, because the train/test split was performed as equally as possible within the natural scene categories, a 0/1 balance in the testing data was not ensured under the naturalness, openness, and roughness conditions. Therefore, to fairly examine the accuracy of the models that classified these global properties, the balanced accuracy calculated using equation (1) was adopted to calculate the classification accuracy for those conditions. Note that tp, fn, fp, and tn denote the numbers of true positives, false negatives, false positives, and true negatives, respectively.


$$\mathrm{balanced}\;\mathrm{accuracy}=\frac{1}{2}\left(\frac{tp}{tp+fn}+\frac{tn}{tn+fp}\right)\ldots \left(1\right)$$

The classification accuracies of naturalness, openness, and roughness were 65.5, 64.9, and 54.4%, respectively (chance level: 1/2 (50%); *t*(10) = 9.57, 10.7, 4.31; *p* = 2.4 × 10^−6^, 8.8 × 10^−7^, 1.5 × 10^−3^ respectively, two-tailed one-sample *t*-test), all of which were statistically significant (*p* < 0.01, FDR-corrected). These results indicate that natural scene categories and global properties can be significantly classified using simple VEPs.

### 3.3. Spatiotemporal maps of VEP components contributing to the classification based on Grad-CAM

**Figure 5:**
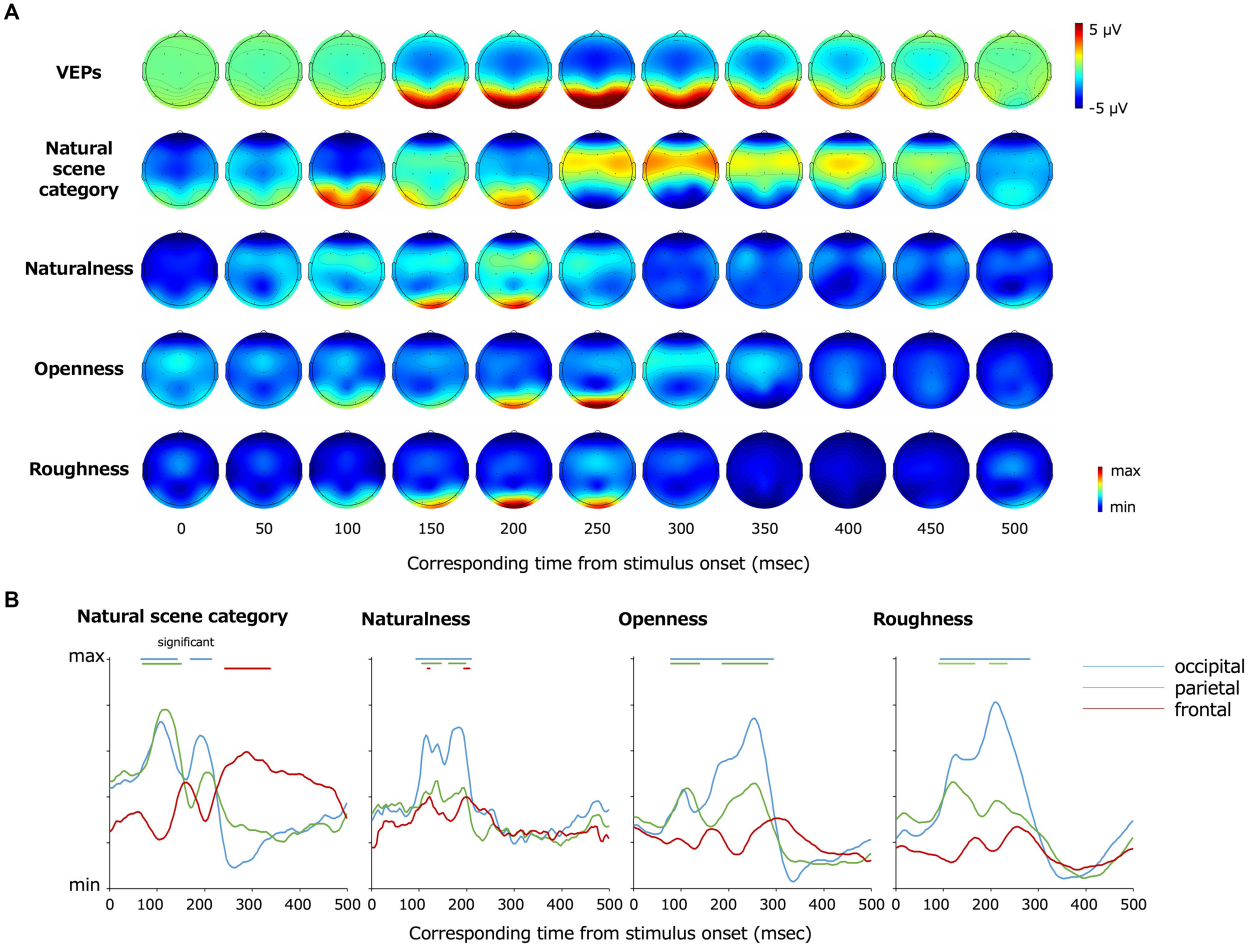
**(A)** Topography of grand average VEPs and VEPs contributing to the classification visualized by Grad-CAM. From the top, the grand average VEPs, the EEG channels that contributed to the classification of the natural scene categories, naturalness, openness, and roughness are shown. **(B)** Re-plot of **(A)** on a plane with the vertical axis representing the degree of contribution and the horizontal axis representing the time from the stimulus onset. The data were averaged for the occipital (O1, O2), parietal (P7, P8), and frontal (F3, F4, F7, F8, Fz, Cz) lobe channels, respectively. The bars on top of each panel indicate the statistically significant periods.

Figure 5A shows the topography of the EEG channels and time points that contributed to the classification visualized using Grad-CAM. The degree of contribution is converted to relative values according to the minimum and maximum values as described in the method section, and we set the minimum values as zero to plot. Statistical analysis was performed by the two-tailed one-sample t-test using the first peak of contribution (the maximum degree of inter-participant averaged contribution within the first 12 time points at all electrodes) in each classification class as a baseline. The multiple comparisons among time points and electrodes were resolved by the BH FDR-correction method with a threshold of *p* = 0.05. Red indicates the maximum contribution to the classification, and blue indicates the minimum. Fp1 and Fp2, which were excluded from the classification, are plotted as making the minimum contributions to the classification. In Figure 5B, the data from the occipital (O1, O2), parietal (P7, P8), and frontal (F3, F4, F7, F8, Fz, Cz) lobes are graphically presented. The data in each panel were averaged across electrodes for visualization.

The occipital and parietal electrodes (O1, O2, P7, P8) at approximately 68–144 and 172–216 ms after the stimulus onset and the frontoparietal electrodes (F3, F4, F7, F8, Fz, Cz) at approximately 244–340 ms contributed to the classification of the natural scene categories.

The frontoparietal electrodes (F3, F4, F7, F8, Fz, Cz) at 120–124 and 196–208 ms after the stimulus onset contributed to the classification of naturalness, and the occipitoparietal electrodes (O1, O2, P7, P8) at earlier and later latencies such as 96–212 ms after the stimulus onset also contributed to the naturalness classification. The occipitoparietal electrodes (O1, O2, P7, P8) at approximately 80–296 ms after the stimulus onset contributed to the classification of openness. Furthermore, occipitoparietal electrodes (O1, O2, P7, P8) at approximately 92–236 ms and occipital electrodes (O1, O2) at approximately ~284 ms after the stimulus onset contributed to the classification of roughness, whereas the other channels did not largely contribute.

These results indicate that the occipital lobe contributed to the classification generally. However, there were certain differences according to the classification classes. As examples, the frontal lobe contributed to the natural scene category and naturalness classification, and the occipital lobe at earlier latencies (approximately 80 ~ ms) contributed to the natural scene category and openness classification.

## 4. Discussion

To investigate the spatiotemporal development of natural scene perception in the human brain, the present study introduced a deep classification model (EEGNet) that classified natural scene categories and global properties by inputting VEPs for natural scene images. As a result, we found that natural scene categories and global properties can be classified at a statistically significant level even using VEPs with low spatial resolution. We also found that the time points and EEG channels that contributed to the classification differed largely depending on the classes of classification. For example, for natural scene category and openness, VEPs in the occipital electrodes at early latencies (approximately 80 ~ ms) contributed to the classification, whereas VEPs in the occipital electrodes at approximately 92 ~ ms mainly contributed to the classification of naturalness and roughness. In addition, VEPs in the frontal and parietal electrodes contributed to the classification of natural scene category, whereas VEPs in the occipital electrodes mainly contributed to the classification of the other classes. These results suggest that the natural scene category is processed in human visual cortex differently from the global properties, and different global properties of natural scenes are processed at different latencies and in different areas of the human brain.

The classification of the 13 individual natural scene categories was supported by the VEPs of the occipitoparietal electrodes at approximately 68–144 and 172–216 ms, and the frontal lobe at approximately 244–340 ms. The contribution of the occipital lobe to the classification of natural scene categories further supports the results of previous studies that revealed the encoding process of natural scenes by VEPs (Scholte et al., 2009; Hansen et al., 2011; Groen et al., 2013; Greene and Hansen, 2020) and is consistent with the idea that scene selective regions such as the occipital place area and retrosplenial complex are distributed in or around the occipital and parietal lobes (Aguirre et al., 1998; Epstein and Kanwisher, 1998; Ishai et al., 1999; O'Craven and Kanwisher, 2000; Julian et al., 2016). In addition, the frontal lobe has been suggested to be associated with natural scene perception, which is consistent with the results of previous studies using fMRI (Peyrin et al., 2004, 2010; Walther et al., 2009). The results also indicate a possibility that natural scene category is processed inter-regionally, from the occipital to frontal lobes.

In terms of naturalness, the classification accuracy was higher than the other classification classes. In addition, the occipital VEPs at relatively early latencies (approximately 96 ~ ms) contributed to the classification. This is not inconsistent with the psychophysical finding that the naturalness of natural scenes can be perceived even with a particularly short presentation time such as 20 ms (Joubert et al., 2007; Greene and Oliva, 2009; Loschky and Larson, 2010). The reason why naturalness is encoded at relatively early latency and accurately classified using the VEPs may be that naturalness can be predicted to some extent by lower-order image features. In fact, another analysis showed that naturalness was predicted with 85.2% accuracy (chance level: 50%; *t*(4) = 11.5; *p* = 3.3 × 10^−4^, two-tailed one-sample *t*-test) by the spatial envelope (Oliva and Torralba, 2001; Torralba and Oliva, 2003) using an SVM, which corresponds to the energy of subbands in spatial blocks.

Furthermore, the fact that the VEPs in the frontal electrodes contributed slightly to naturalness classification supports the results of previous studies using fMRI (Peyrin et al., 2004). This study showed that low-spatial-frequency information was vital for the natural scene perception and conveyed to the right anterior parahippocampal and temporal cortex. In fact, man-made scenes contain many linear contours, whereas natural scenes do not necessarily do so. These differences can also be observed even for low-spatial-frequency information, that is, naturalness can be discriminated by low-spatial-frequency information. Therefore, together with the findings of the previous studies, it is reasonable that the VEPs in the frontal electrodes at approximately 200 ms slightly contributed to the naturalness classification.

The openness classification was supported by the occipital VEPs at early latencies (approximately 80 ~ ms), which is consistent with the previous finding that natural scene openness modulates the P1 component of the ERP (Hansen et al., 2018). This is also consistent with the fact that less than 50 ms presentation of natural scene images allowed openness to be discriminated with high accuracy (Greene and Oliva, 2009). The early encoding of openness may relate to the fact that natural scene openness can be discriminated by lower-order features. In fact, the openness of the visual stimuli was classified with an accuracy of 78.5% (chance level: 50%; *t*(4) = 8.34; *p* = 1.1×10^−3^, two-tailed one-sample *t*-test) by the SD of the spatial frequency subbands (seven scales, one-octave step) of each image using an SVM. The subband SD corresponds to a subset of image statistics known to be important in texture perception in the early visual cortex (Bergen and Adelson, 1988; Heeger and Bergen, 1995; Zipser et al., 1996; Portilla and Simoncelli, 2000; Baker and Mareschal, 2001; Motoyoshi et al., 2007; Freeman and Simoncelli, 2011; Freeman et al., 2013; Ziemba et al., 2019). This image statistic has also been revealed to be strongly correlated with VEPs at as early as 88 ~ ms after the stimulus onset (Orima and Motoyoshi, 2021).

Meanwhile, the classification accuracy of roughness was lower than the other classification classes, and the occipitoparietal VEPs from early to late latency (approximately 92–236 ms) contributed to the classification of roughness. These results support the idea that the roughness is correlated with both lower and higher-order information because roughness started to be encoded early but the classification accuracy was low compared with naturalness and openness. Our analysis showed that the roughness of natural scenes was classified with an accuracy of 61.6% (chance level: 50%; *t*(4) = 4.72; *p* = 9.2×10^−3^, two-tailed one-sample *t*-test) by the cross-spatial-frequency correlation of energy subbands of each image using an SVM. Cross-subband correlations are known as a higher-order image statistic, mainly encoded in V2 (Freeman et al., 2013; Ziemba et al., 2019). Cross-subband correlations have also been revealed to be strongly correlated with VEPs at later latency (150 ~ ms) (Orima and Motoyoshi, 2021), and also correlated with lower-order image statistics such as subband SD. The results in the present study were consistent with these findings.

**Figure 6:**
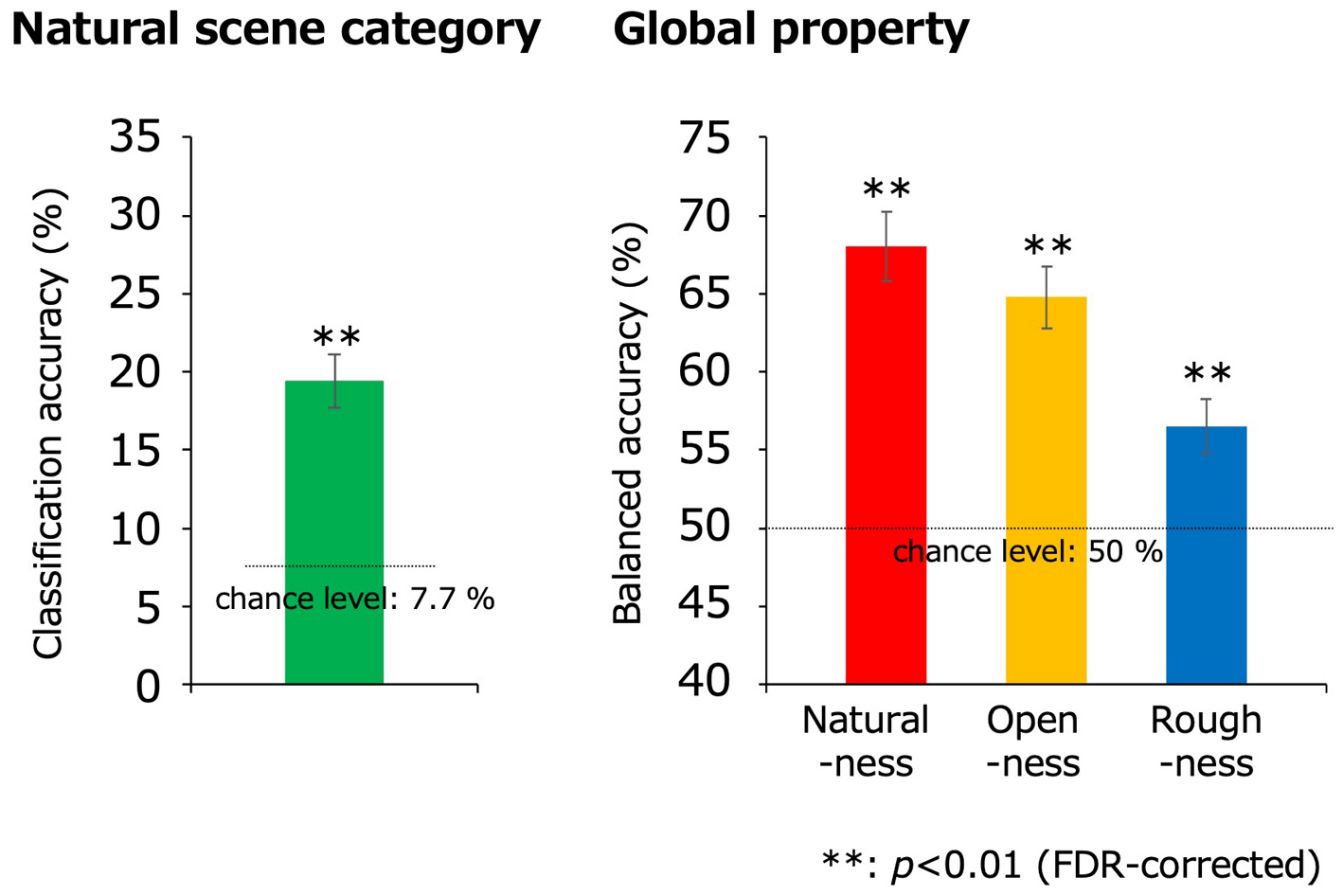
Classification results obtained using the SVM. The green, red, yellow, and blue bars indicate the classification accuracies for the natural scene categories, naturalness, openness, and roughness, respectively, all of which were statistically significant (**p < 0.01, FDR-corrected). The dotted lines denote the chance levels. The error bars indicate ± 1 s.e.m. across observers.

The method we proposed in the present study have a limitation. Figure 6 shows the classification results using SVMs under the same condition as the present study. In fact, the classification accuracy values of the EEGNet models were not higher than those of the traditional SVMs, that is, the EEGNet model did not performed well as a classification model. However, in the present study, we adopted the EEGNet models to visualize the contributing portions of the inputting VEPs by combining with the Grad-CAM. To confirm that the EEGNet model classified global property properly, we trained the EEGNet model to classify roughness, which was originally calculated as continuous values, by using 143 data that had certainly deviated values from the boundary of binarization. As a result, the classification accuracy was 57.6% (*t*(10) = 4.61; *p* = 9.7 × 10^−4^, two-tailed one-sample *t*-test), which was significantly higher than the classification result (54.4%) using all the data (*t*(10) = −2.82; *p* = 1.8 × 10^−2^, two-tailed paired *t*-test). Therefore, the EEGNet model was confirmed to classify roughness correctly. Certainly, we have to mention that there are the other ways to achieve similar goals to the present study such as the sensitivity analysis of the model (Cortez and Embrechts, 2013), the algorithm that interprets the SVMs (Rätsch et al., 2006), and data-driven feature selection methods (Haufe et al., 2014; Kerr et al., 2014). However, the combination of EEGNet models and Grad-CAM enabled us to easily visualize the contribution of input data without repetition of classification analyses for limited EEG channels or latencies, and we can legitimately avoid complicated interpretation of coefficients computed by classification models. In this sense, it is possible that the method in the present study still has certain advantages.

Additionally, there are issues that should be addressed in future studies. First, the EEG preprocess that we applied in the present study could be not the best for the classification by the EEGNet. EEG preprocess consists of which filter to apply to eliminate noises in EEG signals, whether to remove components using ICA or not, and whether to re-reference EEG signals or not, etc. In the present study, we applied typical settings for each of those preprocess steps, but they are desirable to be optimized for the classification if one pursues the highest classification accuracy. In addition, the Grad-CAM results may be changed by the modification of input VEPs because the saliency method can be affected by the slight modification of the input, even if it was a constant vector shift (Kindermans et al., 2019). EEG preprocess definitely involves modification of EEG signals, and there are huge number of variations of EEG preprocessing. In future investigations, it may be desirable to choose optimal parameters for preprocess of EEG signals, taking the shortcomings of saliency methods into account. Second, the architecture of our EEGNet model were the same as those used in the original EEGNet model (Lawhern et al., 2018), but maybe it should be reconsidered using nested-cross validation method to choose appropriate hyperparameters for the EEGNet model. In fact, although our EEGNet models achieved statistically significant classification accuracy for the natural scene categories and global properties, we cannot deny the possibility that the result from the present study, such as the approximate time obtained by Grad-CAM is affected by the EEGNet hyperparameters. For example, in the present study, we used a filter with a kernel size of 64 on the time axis for the first convolutional layer. If a filter with a smaller size were used, the spread of the contribution to the classification shown in Figure 5 might be smaller, and the timing of the maximum contribution might be different. As long as the input EEG data is the same, it is difficult to imagine that the modulation of the hyperparameters, including the filter size, would significantly change the trend of the results. However, in the future studies, it would be preferable to find better hyperparameters for EEGNet models. Third, as a baseline of the classification accuracy, it may be more appropriate to find out chance level by using permutation test (Ojala and Garriga, 2010). The permutation test is performed by the models trained by randomly shuffled labels, which are supposed to fail to acquire the correct relationship between inputs and ground truth labels. This method can also be applied to the sanity check of the Grad-CAM outputs (Adebayo et al., 2018; Farahani et al., 2022).

We have to note that the Grad-CAM itself has certain limitations. According to the previous study that considered the gradient-based attribution methods (Ancona et al., 2019), visualization by the gradient-based methods is strongly affected by high spatial frequency components of input images. For example, edges in input images tend to be regarded as contributing component to classification even if they did not actually. In the present study, inputting VEPs were smooth with respect to the time axis, but not necessarily with respect to channel axis. Therefore, to address shortcomings of the gradient-based methods, it may be desirable to input VEPs that retain the actual channel locations, which are supposed to be spatially smooth. In addition, it is possible that the explanation methods are tricked by the ‘adversarial’ modulation of input images and yield apparently wrong attribution (Dombrowski et al., 2019; Kindermans et al., 2019; Baniecki and Biecek, 2023) as described in the previous paragraph. Absolutely, we did not modify inputting VEPs intentionally, but we cannot deny completely that the results in the present study would change by only a slight modification of the input. Also, we have to mention the baseline of the Grad-CAM. The appropriate baseline are images that has no information such as black images (Ancona et al., 2019) for image classification models. In the present study, we set the baseline, taking the biological validity (VEPs at 0–50 ms after the stimulus onset do not mainly reflect the visual process) into consideration, because we adopted the EEGNet models, whose input was EEG data. However, there are no fixed method for setting baseline and it may be improved in the future studies.

Both psychophysical and physiological studies on scene perception, including the present study, basically use visual stimuli of small size displayed on a conventional computer monitor (e.g., Hansen et al., 2011; Groen et al., 2013). However, given that a goal of scene perception research is to explain our natural scene perception in daily lives, one should ideally use visual stimuli with a sufficiently wide field of view to immerse observers in the scene and allow a high mobility of the observers. It would be difficult to apply such an experimental setting to fMRI experiments that require observers to view stimuli of a limited viewing angle with the head rigidly fixed. In contrast, it may be easier to establish such a free viewing condition with EEG. We expect that the decoding techniques introduced in the present study will also be useful in revealing the cortical dynamics of scene processing in such a natural situation.

## Data availability statement

The raw data supporting the conclusions of this article will be made available by the authors upon reasonable request.

## Ethics statement

The studies involving humans were approved by the Ethics Committee for experiments on humans at the Graduate School of Arts and Sciences, The University of Tokyo. The studies were conducted in accordance with the local legislation and institutional requirements. The participants provided their written informed consent to participate in this study.

## Author contributions

TO and IM designed the research and wrote the manuscript. TO conducted the experiment, developed the DNN models, and analyzed the data. All authors contributed to the article and approved the submitted version.
